# Magnetic study of a mixture of magnetite and metallic iron in indoor dust samples

**DOI:** 10.1007/s11869-016-0412-5

**Published:** 2016-06-09

**Authors:** Beata Górka-Kostrubiec, Iga Szczepaniak-Wnuk

**Affiliations:** Institute of Geophysics, Polish Academy of Sciences, Ks. Janusza 64, 01-452 Warsaw, Poland

**Keywords:** Environmental magnetism, Indoor dust, Metallic iron, Magnetic methods, Atmospheric pollution

## Abstract

Magnetite in mixture with metallic iron in indoor dust samples was examined using several magnetic analyses, thermomagnetic curves of the magnetic susceptibility and the induced magnetization vs. temperature, hysteresis loops, and first-order reversal curves. The study of the magnetic properties was supplemented by the analysis of chemical elements and electron microscopic observation. The metallic iron in the mixture affects the values of hysteresis parameters, decreasing coercivity (*B*_c_) and increasing saturation magnetization (*M*_s_), and it is responsible for the magnetic enhancement of magnetic susceptibility. The thermomagnetic curves show several distinct features: the first Curie temperature of magnetite, the second one (∼764 °C) of iron, and the rapid decrease on the heating curves (between 600 and 750 °C) caused by the oxidation of iron to magnetite. Two magnetochemical processes appear during the thermal treatment of indoor dust: the oxidation of iron to magnetite and the neo-formation of magnetite as a result of chemical transformation of non-magnetic minerals. The shift of the hysteresis parameter ratios from the multi-domain (MD) region towards the single-domain (SD) region on the Day-Dunlop plot is controlled by the oxidation of iron in the thermally induced process and the grain size of the new formed magnetite. The magnetic properties of indoor dust are a potential indicator of indoor air pollution. Elemental iron plays an important role in the development of inflammation in humans via oxidative stress, so that the presence of metallic Fe in indoor dust can affect human health.

## Introduction

During the last decade, a number of experimental and numerical studies advanced the understanding of the emission, formation, dispersion, exposure, and health effect of particulate matter (PM) in urban atmospheres (Kumar et al. 2014; Greenwald et al. 2013; Ayala et al. 2012). On average, people now spend 90 % of their lives inside homes, offices, schools, etc.; the indoor air pollution levels are therefore very important for their health (de Bruin et al. 2008).

Epidemiological studies (Donaldson et al. 1998; Donaldson and Stone 2003; MacNee and Donaldson 2003) try to identify the components of PM, which are responsible for its adverse health effects. For instance, elemental iron is considered to play an important role in the development of inflammation via oxidative stress which is caused by hydroxyl radicals generated during the redox cycling between the Fe(III) and Fe(II) states. The ultrafine, iron-rich particles interacting with cellular components lead to oxidative stress in the fluid of the lung lining (Gilmour et al. 1996; Karlsson et al. 2009).

Numerous studies used magnetometry to detect and monitor airborne pollution (Qiao et al. 2011; Chaparro et al. 2013; Castaneda-Miranda et al. 2014; Revuelta et al. 2014; Shi et al. 2014). Other studies have focused on magnetic properties of indoor dust as a potential indicator of indoor air quality (Górka-Kostrubiec et al. 2014; Górka-Kostrubiec 2015; Szczepaniak-Wnuk and Górka-Kostrubiec 2016).

Górka-Kostrubiec et al. (2014) determined the magnetic characteristics, e.g., mineralogy, grain size, and domain structure, of indoor dust collected from flats and houses in Warsaw, Poland. The histogram of magnetic susceptibility revealed an asymmetric tendency with significant deviation from the normal distribution due to a group of the samples having significantly higher values of magnetic susceptibility. This group of samples evidently differed for its in magnetic mineralogy. Magnetite was the main magnetic mineral, but some samples also contained variable amounts of soft magnetic mineral (metallic iron), which contributed to the magnetic enhancement of the magnetic susceptibility and of the saturation magnetization.

Szczepaniak-Wnuk and Górka-Kostrubiec (2016), evaluating the potential link between different sources of outdoor pollution and indoor air quality, found a correlations between the magnetic susceptibility of indoor dust and heavy metal concentrations. The dust collected from the houses situated in the most polluted parts of the city also revealed high heavy metal pollution levels. Moreover, metallic iron particles were detected in dust from apartments adjacent to high-traffic roads and crossroads in the city center. The authors hypothesized that since the processes associated with the motion of motor vehicles generate Fe-rich particles such as magnetite and metallic iron, their contribution to the magnetic fraction of indoor dust can approximate the impact of traffic-related sources to the indoor air pollution.

The possible interpretation of the presence (abundance) of metallic iron particles as a potential indicator of traffic-related sources motivates us to extend the previous study. In this work, we examined the coexistence of magnetite with metallic iron in indoor dust using the same sets of samples as in Górka-Kostrubiec et al. (2014) and Szczepaniak-Wnuk and Górka-Kostrubiec (2016). Our attention focused on determining the magnetic properties of the magnetite + Fe mixture and those of its thermal product, i.e., after a heating-cooling cycle. The Day-Dunlop plot is commonly used in environmental magnetism for discriminating domain state (single-domain, SD; pseudo-single-domain, PSD; multi-domain, MD) and, by implication, grain size (Dunlop 2002a, b), so it is important to explain how contribution of metallic iron controls the distribution of the hysteresis parameter ratios on this plot.

The magnetic properties of indoor dust can reflect the means of penetration of pollutants into apartments, and on the basis of morphology and the very specific composition of the magnetic fractions, it is possible to indirectly reveal the origin of the pollution sources. For example, the positive correlations of Ca/Fe and Mg/Fe show that their possible source is vehicle traffic as Ca and Mg elements are components of detergent additives added to common engine oil (Monaci et al. 2000). Moreover, Ni and V elements are derived mainly from fossil fuel emissions, Ba is a common component in automobile break pads, and Cr, Mn, Pb, and Zn are currently the main metallic pollution emitted by motor vehicles. Their concentrations very well correlates with the magnetic parameters depending on the concentration of magnetic particles in indoor dust Szczepaniak-Wnuk and Górka-Kostrubiec (2016).

## Methods and measurements

### Study sites and sample collection

Indoor dust samples taken from 200 flats or houses in Warsaw, Poland, and from 20 flats from the small town Zyrardow situated in Central Poland 45 km southwest of Warsaw were examined in the study. The sampling sites were distributed in the city center, in areas with heavy traffic, and in low-polluted suburbs. The residents of apartments filled in a form containing questions about the apartment localization (distance from the main roads, green areas, and type of public transport) and the customs of inhabitants (cigarette smoking, using home heaters, cooking, animals, etc.).

Dust samples from Warsaw were collected in the same period between 1 April and 15 May 2011 and the second time between 1 September and 15 October 2011. In Zyrardow, the dust was gathered over the course of one mount on September 2014. The samples did not show dependence on the sampling periods (seasons) as in the Polish climate, they do not differ in meteorological conditions.

The indoor dust was collected by owners of apartments using vacuum cleaners with the multi-layer bags. The cleaning of floor surface of apartment and furniture was equivalent to collecting the samples. In a laboratory, dust was removed from the vacuum cleaner bags, mechanically sieved through a mesh with 1 mm openings, and then packed into plastic boxes. Sieving removes large pieces of food, paper, toys, animal dander, hair, etc. The homogeneous powder (about 1 kg of mass) was used to examine magnetic properties and for microscopic and chemical analysis.

A detailed description of the sampling procedure can be found in previous works, e.g., Górka-Kostrubiec et al. (2014) and Górka-Kostrubiec (2015).

### Methods and instruments

The low-field magnetic susceptibility (*κ*) was measured twice for each sample using the Multi-Function Kappabridge (MFK1-FA, AGICO), with frequencies of 976 and 15,600 Hz, sensitivity of 2 · 10^−8^ SI, and magnetic field (*H*) of 200 A m^−1^. The mass specific magnetic susceptibility (*χ*) was calculated by dividing *κ* for the mass of the sample. The frequency-dependent value of magnetic susceptibility (*χ*_fd%_) was computed as the percentage of magnetic susceptibility loss measured at low and high frequencies of the applied magnetic field.

For samples before heating and after cooling, the hysteresis loops, the isothermal remanent magnetization (*IRM*) acquisition, and its backfield demagnetization curves were measured with an advanced variable field translation balance (AVFTB; Petersen Instruments, Germany). The saturation remanence (*M*_rs_), the saturation magnetization (*M*_s_), and the coercivity (*B*_c_) were determined from the hysteresis loops (the maximum applied field of 1 T) and the coercivity of remanence (*B*_cr_) from the curve of subsequent DC backfield demagnetization of *IRM*. The mass-normalized values of *M*_s_ and *M*_rs_ after correction of the hysteresis loop for paramagnetic fraction were calculated.

The thermal changes of induced magnetization (*M*(*T*)) and magnetic susceptibility (*κ*(*T*)) are the main magnetic features for quickly characterizing the magnetic mineralogy according to the estimation of the Curie temperatures. The change in *M*(*T*) depends mainly on ferromagnetic phase composition and crystal structure. The magnetic susceptibility (*κ*(*T*)) varies in a more complicated manner; it is dependent on composition and also on thermally induced chemical transformations, occurring in minerals, and grain size (Thompson and Oldfield 1986). Magnetic minerals were recognized by the characteristic temperature of their magnetic transformation to paramagnetic phase, i.e., the Curie temperature, which was estimated from the curves of *M*(*T*) and *κ*(*T*). The temperature obtained for the inflection point on the curve’s steepest decline was taken as the Curie temperature. For calculation of the inflection point, the second derivative method was applied (Petrovský and Kapička 2006).

The curves of *κ*(*T*) were measured with a Multi-Function Kappabridge (KLY-3, AGICO) coupled with a CS-3 high-temperature furnace. These measurements were conducted in the range from room temperature up to 700 °C, in an air atmosphere. The curves of *M*(*T*) were measured from room temperature up to 800 °C, in an air atmosphere, using the AVFTB. The induced magnetization was measured in the low magnetic field (*H*) of 28 kA m^−1^ and in the magnetic field of 400 kA m^−1^ which saturated *M*. The temperature experiments were performed on powder samples of indoor dust, on their magnetic extracts (magnetic fraction of dust), and on the fraction remaining after separation using a neodymium hand magnet.

First-order reversal curves (FORCs) were measured using the alternation gradient magnetometer (AGM; MicroMag, Princeton Measurement Corporation, USA). FORCs were measured with typical settings: increments of 2.0 mT, an average time of 150 ms, and a saturation field of 500 mT (Roberts et al. 2000). Processing, smoothing, and drawing of the FORC diagram were performed according to FORCinel (Harrison and Feinberg 2008), an IGOR Pro program.

The evaluation of the morphology and shape of magnetic particles was performed using scanning electron microscopy (SEM). The SEM observation was supplemented by analysis of chemical composition using energy-dispersive X-ray spectroscopy (EDS). The concentrations of chemical elements in indoor dust were measured using inductive plasma coupled mass spectrometry (IPC-MS; ELAN 6100 DRC from PerkinElmer). Microscopic observation and analysis of chemical composition were carried out in the standardized laboratory of the Faculty of Geography and Regional Studies of the University of Warsaw.

In order to find the contribution of several heavy metals to the overall toxicity status of dust samples, the Tomlinson Pollution Load Index (PLI) was used (Tomlinson et al. 1980). The PLI is defined as the *n*th root of the product of the concentration factors (CF_*n*_)

1 $$ \mathrm{P}\mathrm{L}\mathrm{I}=\sqrt[n]{{\mathrm{CF}}_1\cdot {\mathrm{CF}}_2\dots {\mathrm{CF}}_n},\kern0.5em \mathrm{where}\kern0.5em {\mathrm{CF}}_n=\frac{C_n}{C_{n\left(\mathrm{background}\right)}} $$

The CF_*n*_ is a ratio of the concentration of a heavy metal (*C*_*n*_) to the corresponding background value obtained for a reference site. The PLI is approximately 1 if the elemental load is near the background level and greater than 1 if the environment is exposed to metal toxicities (Qiao et al. 2011). It is very difficult to establish a reference site for collecting indoor dust because of the influence of different outdoor and indoor sources. Therefore, the lowest concentrations (*C*_*n*(background)_ = *C*_*n*(min)_) measured for each heavy metal were adopted as background values. In this manner, the PLI shows how much the concentration of heavy metals for a particular sample exceeds the minimum value. For indoor dust, the PLI was calculated for the following heavy metals: As, Ba, Cd, Co, Cr, Fe, Mn, Ni, Pb, and Zn, which in the literature are very often considered as traffic-related.

## Results and discussion

**Fig. 1:**
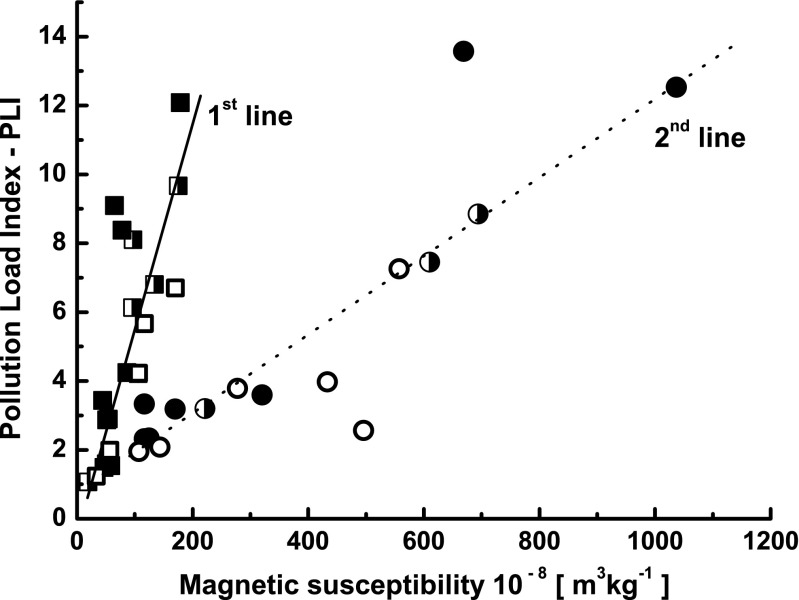
Correlations between magnetic susceptibility (*χ*) and concentration of heavy metals expressed by the Tomlinson Pollution Load Index (PLI). The data marked by *open symbols* (*open square* and *open circle*) were obtained in this work, the data marked by *half-open symbols* (*half-open square and half-open circle*) were taken from Górka-Kostrubiec et al. (2014), and the data marked by *closed symbols* (*closed square* and *closed circle*) were taken from Szczepaniak-Wnuk and Górka-Kostrubiec (2016)

Our previous work (Górka-Kostrubiec et al. 2014) presented detailed magnetic characterization of indoor dust, morphology of magnetic particles, and their chemical composition. Figure 1 shows the correlation between magnetic susceptibility and PLI. The open symbols represent data for the dust collected in Warsaw and measured in that work. The data marked by half-open symbols, also from Warsaw, are taken from Górka-Kostrubiec et al. (2014). The closed symbols represent the data for indoor dust from Zyrardow after Szczepaniak-Wnuk and Górka-Kostrubiec (2016). In Fig. 1, two different linear correlations between *χ* and PLI are visible; the second line (Pearson correlation coefficient (*R*) = 0.89, significance level *p* < 0.0001) is relative to the samples showing significant magnetic enhancement of *χ*.

The magnetic susceptibility is influenced by the concentration of magnetic particles, their mineralogy, and the contribution of ultrafine superparamagnetic (SP) grains. Therefore, the magnetic enhancement of *χ* observed in the second group of samples may be due to an increase in the concentration of ferromagnetic/ferrimagnetic minerals, like magnetite, maghemite, and iron, and/or an increase in the contribution of SP particles. Our previous studies have shown that the *χ*_fd%_ values of indoor dust are in the narrow range between 1 and 3.5 %, indicating an insignificant amount of SP particles (Dearing et al. 1996). For this reason, our interest was focused on the differences in mineralogy between samples of the second group and the samples approximated by the first line in Fig. 1 (the first group). The magnetic fraction of the first group samples primarily consists of magnetite. This follows from the Curie temperature of magnetite, ∼585 °C, observed on the heating and cooling curves of *κ*(*T*) and the hysteresis loops typical of magnetite with *B*_c_ of 7–9.5 mT and B_cr_ of 26–28 mT (Górka-Kostrubiec et al. 2014). Our result is consistent with the study of the urban PM magnetic fraction, mainly containing magnetite with hematite as a secondary magnetic phase in industrial contexts (Muxworthy et al. 2002; Zheng and Zhang 2008; Zhang et al. 2010; Magiera et al. 2011; Qiao et al. 2011; Sagnotti and Winkler 2012).

### Magnetic mineralogy of indoor dust samples showing high magnetic enhancement of magnetic susceptibility

**Fig. 2:**
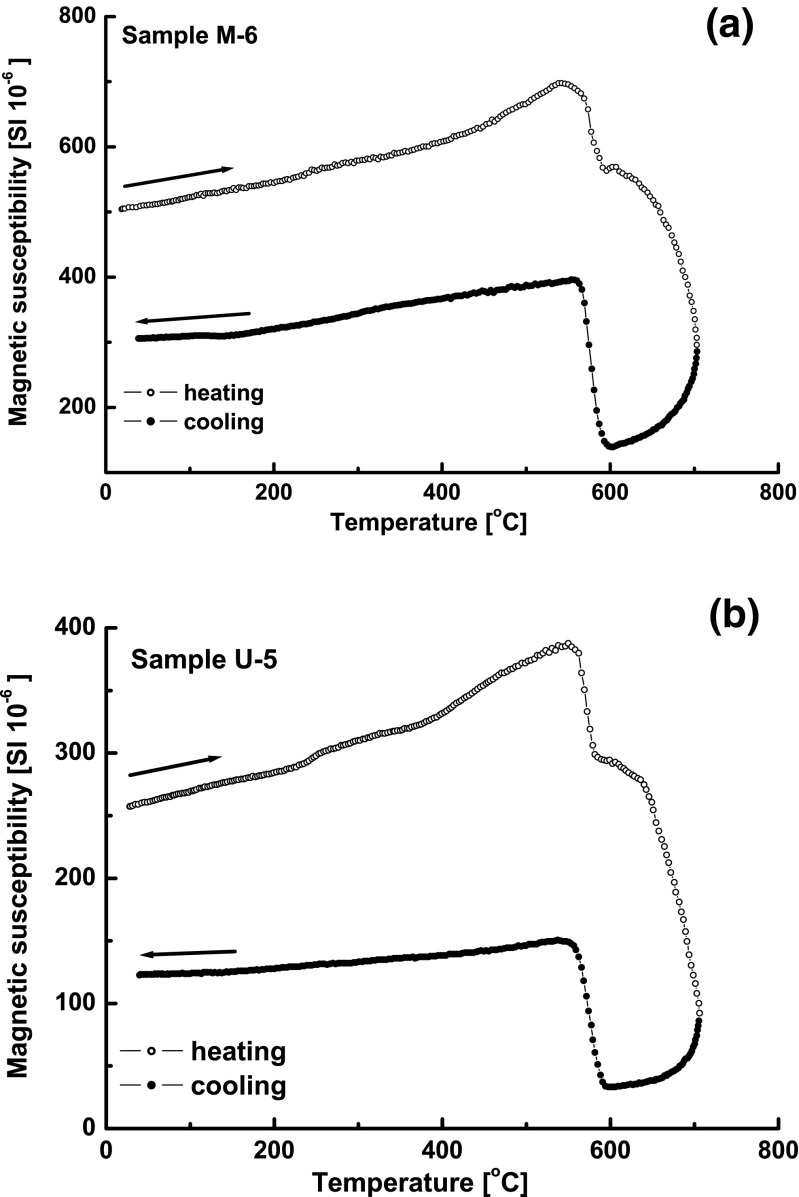
Temperature dependence of magnetic susceptibility (*κ*(*T*)) for the selected samples from the second group: **a** M-6 and **b** U-5. Heating curve (*open circles*) and cooling curve (*closed circles*)

**Fig. 3:**
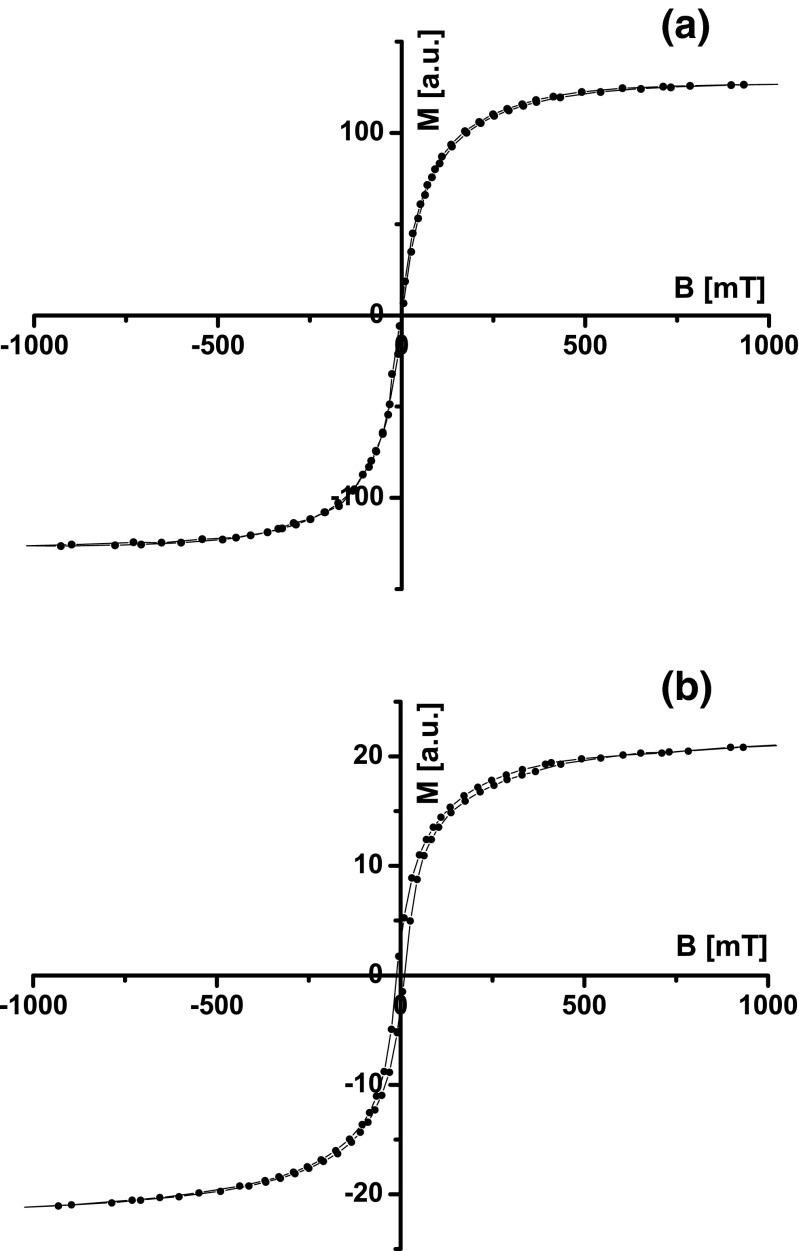
The hysteresis loops for M-6 (**a**) and C-15 (**b**) samples. The hysteresis loops are before correction of paramagnetic fraction

Figure 2 shows the curves of *κ*(*T*) for two representative samples from the second group. The heating curves show a weak increase of *κ* with rising temperature and the Hopkinson peak occurring just before the Curie temperature (*T*_CM_) of magnetite, estimated at about ∼585 °C. In the temperature range between 600 and 700 °C, *κ* declines very rapidly and does not reach the low values expected for the paramagnetic phase. During cooling, the decline continues until the *T*_C_ of magnetite. The estimated Curie temperature, ∼585 °C, confirms that magnetite is a dominant magnetic mineral in the samples of indoor dust. The behavior of *κ* in the temperature range from 600 to 700 °C (Fig. 2a, b) may be indicative that the samples of the second group, in addition to magnetite, contain magnetic mineral with Curie temperatures above 700 °C, such as metallic iron or iron alloys. The very narrow hysteresis loops (Fig. 3) with the high values of *M*_s_ (169–1990 · 10^−3^ Am^2^ kg^−1^) and low values of *B*_c_ (3–5.5 mT) and *B*_cr_ (10–25 mT) suggested the magnetite mixed with soft magnetic minerals, probably iron.

**Fig. 4:**
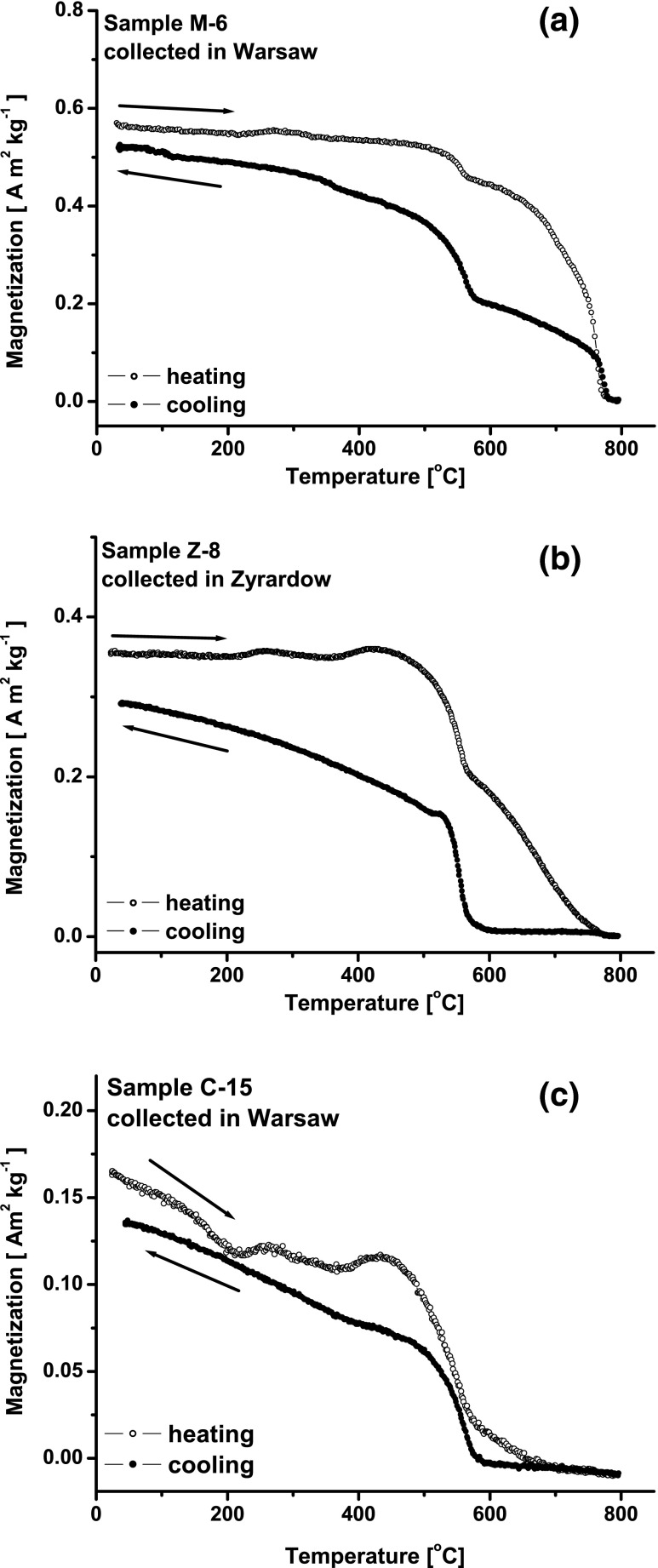
Temperature dependence of induced magnetization (*M*(*T*)) for the selected samples from the second group: M-6 (**a**), Z-8 (**b**), and C-15 (**c**). Heating curve (*open circles*) and cooling curve (*closed circles*)

In order to determine which additional magnetic mineral is present in the second group of samples, the variation of induced magnetization (*M*(*T*)) in the temperature range from 20 to 800 °C was measured. Figure 4 shows the curves of *M*(*T*) for two samples (M-6 and C-15) from Warsaw and for one sample (Z-8) from Zyrardow. The measurement was conducted in a saturation magnetic field (*H*) of 400 kA m^−1^. All the curves of *M*(*T*) show a magnetic transformation of magnetite similar to that observed on the curves of *κ*(*T*). Above 600 °C, the magnetization still decreases with the increasing temperature and, finally, the second magnetic transition is reached at a Curie temperature (*T*_CFe_) of ∼760–765 °C, which can be attributed to metallic iron or iron-based alloys. For all samples, the magnetic enhancement is visible because the cooling curves are below the heating curves (Fig. 4a–c). Investigating the magnetite phase produced by ball milling of hematite with iron (environmentally polluted samples containing hematite and iron), Petrovský et al. (2000) showed that heating in air causes oxidation of Fe and neo-formation of magnetite. The authors concluded that the high values of *κ* above magnetite’s *T*_C_, visible as the decreasing tail on the heating curve of *κ*(*T*), are an indirect proof of the presence of unoxidized metallic iron.

The decrease in magnetization between 600 and 750 °C observed for indoor dust may be associated with the transformation of metallic iron to magnetite. It is effectively visible in the cooling curve, going backwards from 800 to 580 °C (Fig. 4a–c). The magnetization is lower, in this interval, because metallic iron has been oxidized into magnetite, which is paramagnetic up to 580 °C. The M-6 has high magnetization in the cooling curve even between 800 and 580 °C (Fig. 4a), probably indicating that not all the magnetic iron is yet oxidized. For C-15 (Fig. 4c), having a relatively low-mass magnetic susceptibility (Fig. 1) in the second group, the decline in *M*(*T*) above 600 °C is weaker than that in M-6 and Z-8. The magnetization is close to zero just above 700 °C (Fig. 4c), probably because of the thermal energy, which was sufficient to complete oxidation of the small amount of iron and/or because C-15 has the more complex mineralogy, a mixture of iron oxides. Based on the intensity of changes of magnetization above 600 °C, it could be possible to quantify the amount of metallic iron in a sample. However, particle size should be considered as the small grains are an additional factor accelerating the oxidation process, resulting in a faster decrease of *M* at high temperatures.

**Fig. 5:**
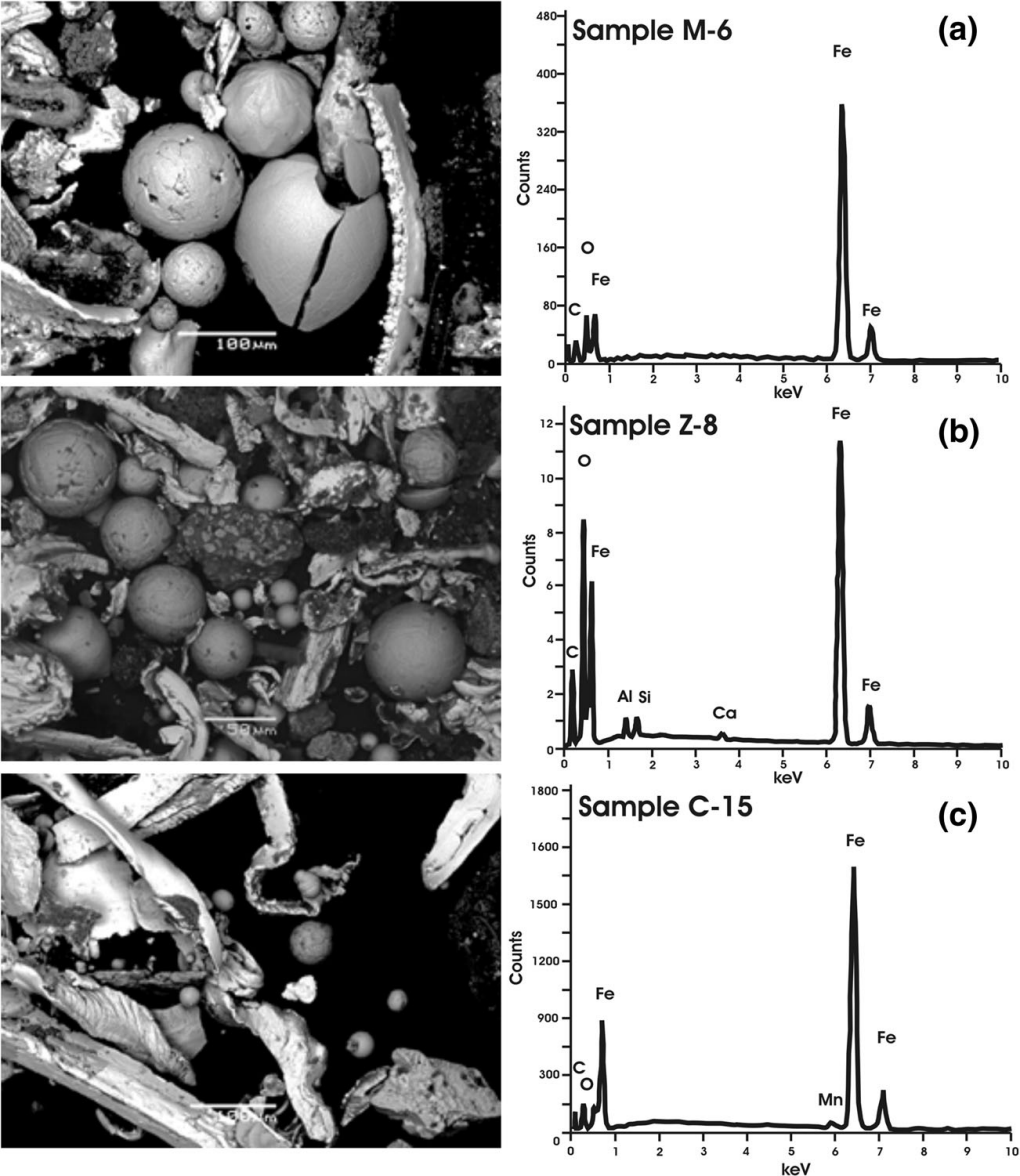
Scanning electron microscopy (SEM) images with energy-dispersive X-ray spectroscopy (EDS) spectra for the magnetic extract of indoor dust from Warsaw, M-6 (**a**) and C-15 (**c**), and from Zyrardow, Z-8 (**b**)

SEM observations in conjunction with EDS analysis were used to identify the Fe-rich particles in indoor dust from the second group of samples. The SEM images of the magnetic extract revealed two major types of particles, spherules and irregular-shaped grains, among which, elongated, shaving-like particles and chip-like particles (Fig. 5) have been seen. The spherules differ in surface morphology (orange-peel, hexagonal-pattern, thread-like, and druse-like) and diameter, ranging from several to more than 200 μm. The EDS analysis of orange-peel and hexagonal-pattern spherules revealed mainly Fe with minor Ca content. Elongated and chip-like magnetic particles observed in all samples of the second group were composed of Fe, O, and C, their diameters ranging from 10 to 600 μm because of their irregular shapes.

Characteristic of the magnetic particles of indoor dust is also supported by the study of Zhang et al. (2012), performed on street dust collected from the area of a Fe-smelting plant. The high values of *χ* (>1000 · 10^−8^ m^3^ kg^−1^), the low coercivity values (3–9 mT), and the decreasing tail visible on the heating curve of *k*(*T*) above 600 °C confirm the presence of iron in street dust. The authors concluded that the magnetic grains were coarser in size because of the distribution of hysteresis parameters on the Day-Dunlop plot in the region of about 90–100 % MD contribution. Fe-rich particles were detected in PM collected on air filters in an urban area and in street dust from a fast-developing industrial zone by Muxworthy et al. (2002). Based on the Mössbauer spectra and magnetic measurement of urban atmospheric PM, they found magnetite in grain sizes between 0.1 and 0.7 μm with a significant amount of iron. Magnetite, the primary magnetic component, came from automobiles, whereas iron particles came directly from street trams, which ran near the sampling locations. Zheng and Zhang (2008) established the link between iron particles in street dust and high traffic density in urban areas. The origin of the iron particles was reasonably associated with their emission through abrasion of the combustion cylinders, pads, and disc brakes of vehicles. Metallic iron was not found in topsoil because of its fast conversion into other iron-containing minerals by redox and/or other chemical reactions with organic matter. The physical and morphological properties of street dust from the high-density traffic area with the Fe-smelting plant revealed the presence of anthropogenic magnetic spherules, iron particles from the plant, and traffic-related angular-shaped particles (Zhang et al. 2012). Different heavy metals were found to originate from the different anthropogenic activities: Fe, Co, and Mo from the Fe-smelting plant; Cu and Ni from vehicle traffic; and Pb, Zn, and Cd from both sources.

### Magnetic characteristic of magnetite and metallic iron mixture and of its thermal product

The result of the thermomagnetic analysis indicates two processes taking place simultaneously in indoor dust: the first process is related to the oxidation of metallic iron resulting in the production of magnetite, and the second one is connected to the formation of magnetite as a product of chemical transformation of non-magnetic minerals. The newly formed magnetite, the final thermal product of both processes, affects magnetization, but its amount depends on both the content of metallic Fe and the chemical composition of the indoor dust.

**Fig. 6:**
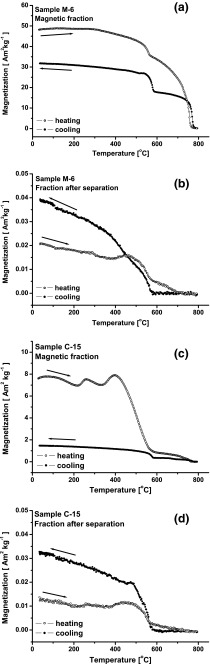
Temperature dependence of magnetization (*M*(*T*)) for magnetic fraction of M-6 (**a**) and C-15 (**c**) and for fraction remaining after separation of M-6 (**b**) and C-15 (**d**). Heating curve (*open circles*) and cooling curve (*closed circles*)

Further detailed analysis was performed after the separation of dust samples into two fractions: the first fraction called “magnetic fraction” consisted of mainly metallic iron and magnetite, and the second fraction remaining after separation called “fraction after separation” contained residual magnetite and Fe-rich particles due to separation and/or hard magnetic minerals like hematite and other non-magnetic minerals and was not perfect. In this manner, it should be possible to discriminate magnetite due to the iron oxidation from that produced during the thermal alteration of the non-magnetic fraction of the dust. For the magnetic fraction, the heating and cooling curves of *M*(*T*) (Fig. 6a, c) show two Curie points: the first for magnetite and the second for iron. For both samples, the heating curves reveal magnetic enhancement compared to the cooling curves. Iron is known to have a higher magnetization than magnetite; thus, the oxidation of Fe in the thermal process causes the increase of the magnetite concentration and the decrease of magnetization shown on the cooling curve. Consequently, the cooling curve is below the heating one.

The heating curves of the fraction remaining after separation (Fig. 6b, d) show mainly magnetite and a small portion of metallic iron which is almost completely oxidized to magnetite just below the *T*_CFe_. The cooling curves show only the *T*_CM_ (Fig. 6b, d) and a relatively high magnetic enhancement compared to the heating curves. This result may indicate that the formation of new magnetite via chemical transformation of non-magnetic compounds of dust is initiated by thermal energy during heating. This effect is very low compared to the intensity of process occurring in magnetic fraction. The effective increase in magnetization on the cooling curve is from 0.02 to 0.04 Am^2^ kg^−1^, while for the magnetic fraction, the effective decrease in magnetization is from 50 to 30 Am^2^ kg^−1^. Our result can be supported by the study of dust collected at the edges of roads in three typical industrial cities in China (Zhu et al. 2013). The increase of *κ* with a temperature up to 500 °C was interpreted as neo-formation of magnetite as a result of thermal alteration of Fe-rich clay minerals, which was confirmed by the significant increase of *κ* values on the cooling curve for a temperature <500 °C.

### Influence of metallic iron on the distribution of hysteresis parameter ratios on the Day-Dunlop plot

**Fig. 7:**
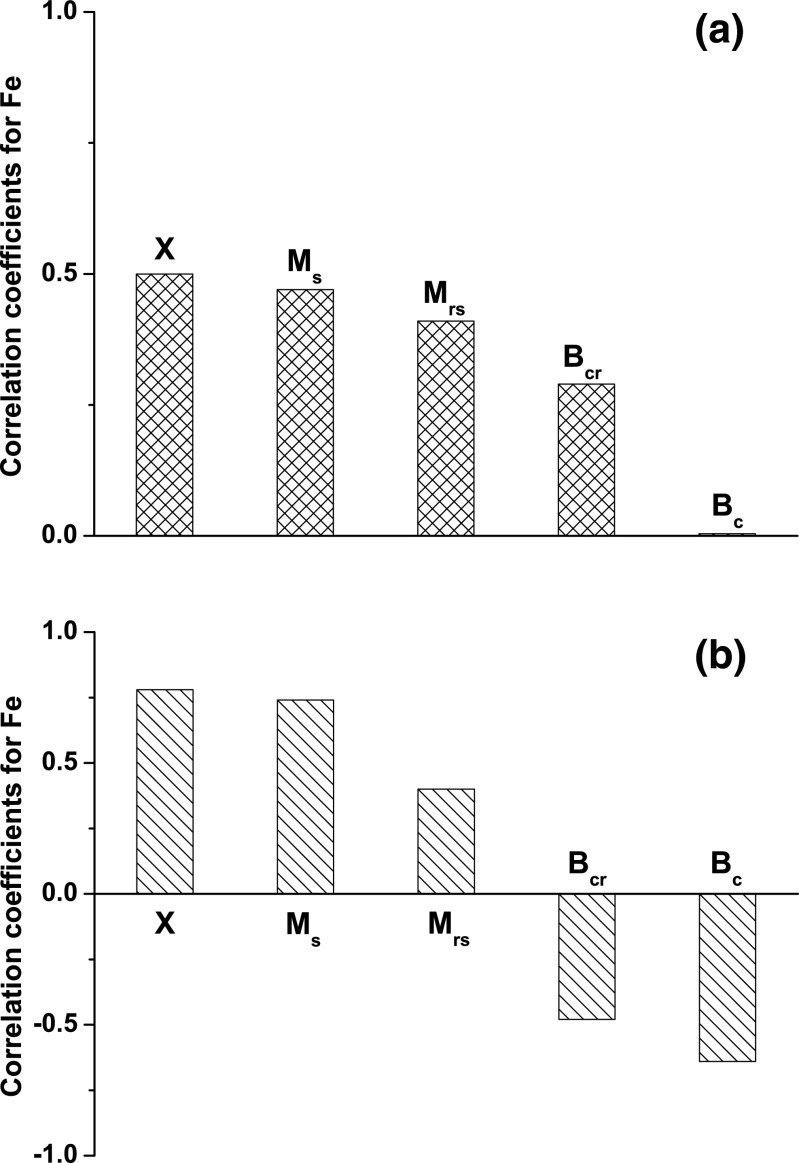
Correlations of concentration of elemental Fe with magnetic parameters: mass magnetic susceptibility (*χ*), saturation magnetization (*M*_s_), saturation remanence (*M*_rs_), coercivity (*B*_c_), and remanence coercivity (*B*_cr_), **a** for the first group of samples approximated by the first line in Fig. 1 and **b** for the second group of samples approximated by the second line in Fig. 1

**Fig. 8:**
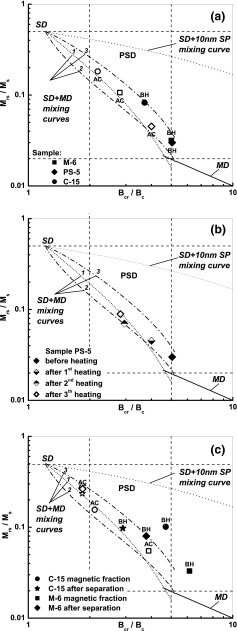
Hysteresis loop parameter ratios on the Day-Dunlop plot for the selected samples of the second group: data for M-6, PS-5, and C-15 before heating (*closed marks*, *BH*) and after a heating-cooling cycle (*open marks*, *AC*) (**a**); data for PS-5 after three subsequent heating-cooling cycles (**b**); and data for magnetic fraction and fraction remaining after separation (**c**). The single-domain (*SD*), pseudo-single-domain (*PSD*), and multi-domain (*MD*) regions and the theoretical mixing curve for SD + MD, MD, and SD + 10 nm SP grains of magnetite are marked after Dunlop (2002a, b)

Zhang et al. (2010) reported that in a sample dominated by hematite, even a small quantity of pure iron influences the parameters of the hysteresis loop. We suspected that in the sample dominated by magnetite, the presence of the soft magnetic mineral like metallic Fe can also impact the hysteresis loop parameters. Figure 7 shows how the concentration of iron affects the concentration-dependent parameters, *χ*, *M*_s_, and *M*_rs_, as well as the coercivity-dependent parameters, *B*_c_ and *B*_cr_. The correlations between the magnetic parameters and the concentration of elemental Fe, included in all the iron-containing compounds of indoor dust, were calculated using the statistical package “Statistica.” Pearson coefficients with significance levels below 0.01 were computed separately for both groups of samples: for 21 samples from the first group and for 15 samples from the second group. It was found that in the first group of sample, the concentration of elemental Fe correlates positively with all the parameters but very weak with *B*_c_ (Fig. 7a). For the samples with variable amounts of metallic iron, the concentration of elemental Fe correlates negatively with the parameters *B*_c_ and *B*_cr_ and positively with the parameters χ, *M*_s_, and *M*_rs_ (Fig. 7b). This allows us to hypothesize that the magnetic enhancement of *χ* and *M*_s_ in the magnetite + Fe mixture mainly comes from the contribution of soft magnetic iron particles, which simultaneously decreases *B*_c_ value. Consequently, the distribution of hysteresis parameter ratios on the Day-Dunlop plot, which was designed to determine the magnetic domain structure of magnetite grains (Day et al. 1977; Dunlop 2002a, b), should be modified for magnetite-iron mixture. According to this hypothesis and to the trends in Fig. 7, we expect that the ratio of *B*_cr_/*B*_c_ shifts upward since the increase in iron content decreases *B*_c_ more than *B*_cr_ and the ratio of *M*_rs_/*M*_s_ shifts downward since the increase in iron content increases *M*_s_ more than *M*_rs_ compared to a sample comprising only magnetite. In order to verify this hypothesis, the hysteresis loops for M-6, PS-5, and C-15 before heating and after a heating-cooling cycle were determined. On the Day-Dunlop plot (Fig. 8a), the samples before heating have lower values of *M*_rs_/*M*_s_ and higher values of *B*_cr_/*B*_c_ compared to the values after the heating-cooling cycle. Initially, the data for M-6 and PS-5 cluster near the MD area, while the data for C-15 lies in the central region of the plot, near the third SD + MD mixing curve. After heating, all the data shift towards the region of SD grains, confirming the previous hypothesis (Fig. 8a).

**Fig. 9:**
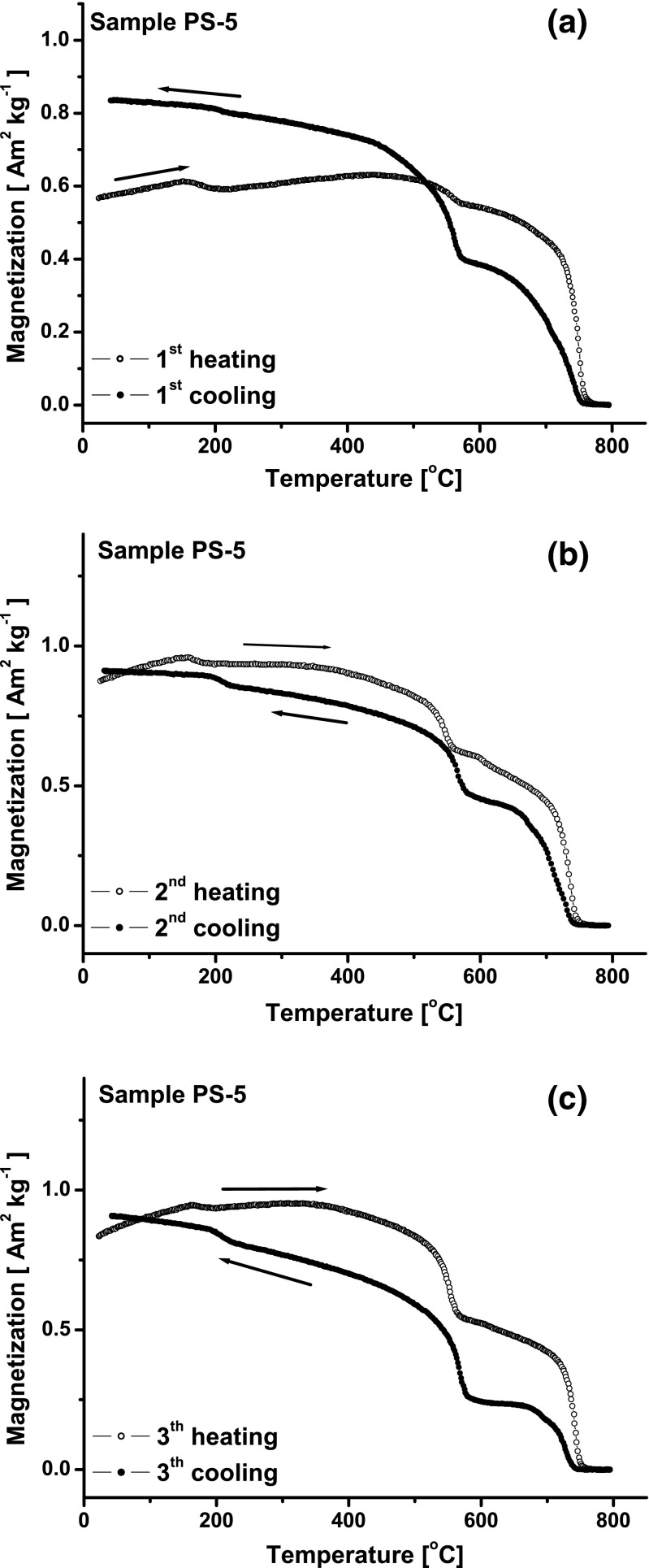
Temperature dependence of magnetization (*M*(*T*)) for PS-5 after three subsequent heating-cooling cycles. Heating curve (*open circles*) and cooling curve (*closed circles*)

The dynamics of the Fe oxidation process and the relocation of the hysteresis parameter ratios on the Day-Dunlop plot are visible in Figs. 8b and 9. For PS-5, the three subsequent curves of *M*(*T*) (Fig. 9) show that only some metallic iron is oxidized to magnetite during the first heating-cooling cycle and the oxidation process is continued in the next two cycles. Consequently, on the Day-Dunlop plot, the first heating-cooling cycle causes the upward shift of the *M*_rs_/*M*_s_ ratio and the downward shift of the *B*_cr_/*B*_c_ ratio (Fig. 8b); in the next two, heating leads to further change of these ratios’ positions in the same direction. The effect is mainly associated to the reduction of the amount of iron during its thermal oxidation, however, and the grain size of the newly formed magnetite can also affect.

Figure 8c shows the distribution of the ratios of hysteresis parameters on the Day-Dunlop plot for the magnetic fraction and for the fraction remaining after separation. The analysis of both fractions severally allows to track the changes in hysteresis parameters caused by thermal oxidation of Fe, which predominantly occurs in the magnetic fraction. On the other hand, the second fraction, which has minor importance in indoor dust, gives information about the grain size of new magnetite created through chemical transformation of non-magnetic minerals. For the fraction remaining after separation, the data before heating is located in the central region of the plot, between the first and third SD + MD mixing curves (Fig. 8c). As seen for the complete samples (Fig. 8a), the data of the thermal product (after heating and cooling) move towards the SD region. This is indicative of the formation of relatively finer magnetite. For the magnetic fraction, the data before heating is above the SD + MD mixing curves, probably because of relatively high values of *B*_cr_/*B*_c_. The thermal product is located between the first and third SD + MD mixing curves. In summary, the hysteresis ratios of the thermal product are located in different areas on the Day-Dunlop plot for both fractions.

### FORC diagram for indoor dust sample

**Fig. 10:**
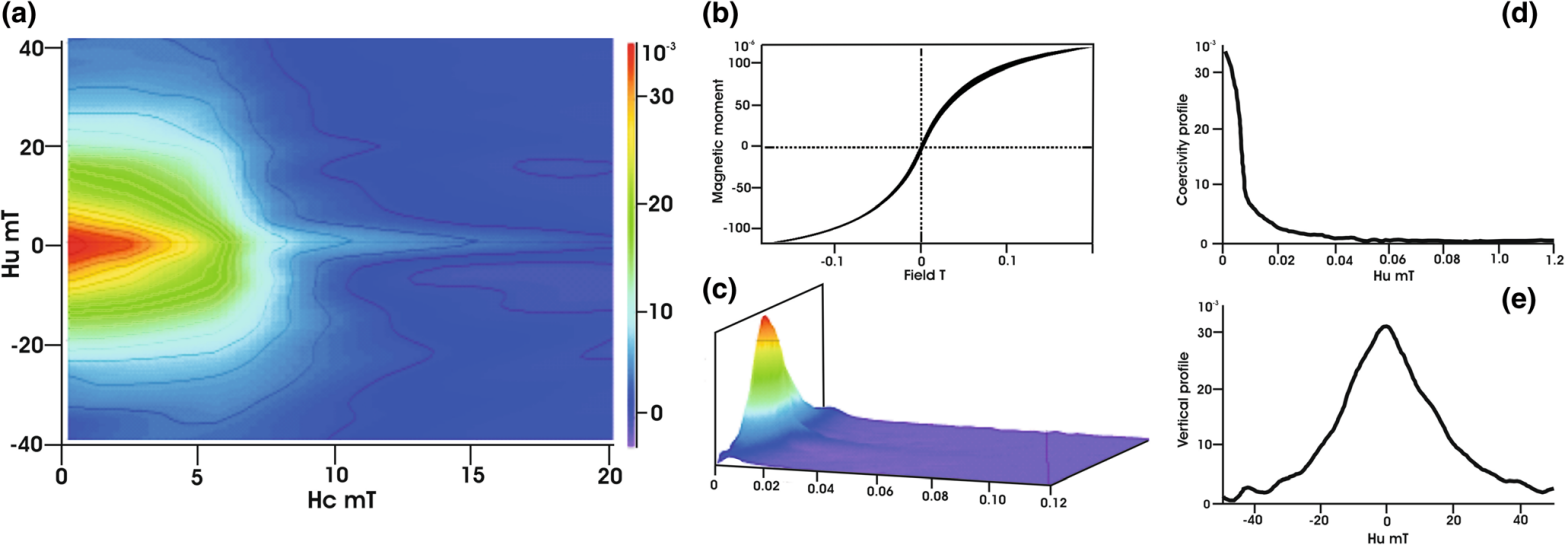
FORC diagram for the magnetic fraction of the M-6 sample. FORCinel and IGOR Pro program (Harrison and Feinberg 2008) was used to compute the following: **a** 2D diagrams, **b** hysteresis loops, **c** 3D diagrams, **d** horizontal profiles, and **e** vertical profiles

FORC is used to investigate the magnetic interactions, the coercivity distribution, and the domain state by measuring partial hysteresis loops (Roberts et al. 2000). For the magnetic fraction of M-6, the FORC diagram in 2D and 3D projection, with horizontal *B*_c_ profile and vertical *B*_u_ profile, is presented in Fig. 10. Pike et al. (2001) studied several materials, among them is transformer M80 steel which is an extremely soft magnetic material with the coercivity of *B*_c_ <1 mT and its magnetization is almost entirely reversible. Transformer M80 steel has a FORC diagram consisting of vertical contours and FORC distribution function that decreases with increasing *B*_c_. Sagnotti and Winkler (2012) and Sagnotti et al. (2009) investigated the magnetic characterization of particles in traffic-related PM, i.e., samples collected from gasoline exhaust, from the leaves of trees in Rome, and powder from rims of car’s front wheels, close to disc brakes. The FORC diagram for the gasoline sample resembled the mixture of low-coercivity MD + PSD grains, while the leaf and brake samples had FORCs typical of MD- and SP-rich grains. Based on the comparison of the FORC distributions for transformer steel and traffic-related dust with those for indoor dust, one can assume that the contribution of metallic iron is responsible for the flattening of the vertical contour of the FORC diagram. Therefore, in indoor dust, the contribution of metallic Fe may be responsible for the shift of all the contours to lower values of coercivity. The diverging contours on the FORC diagram (Fig. 10) indicate that the magnetic fraction of the M-6 contains a MD grain size component, but a minor PSD grain size component may also be present.

### Thermally activated magnetochemical processes occurring in indoor dust samples

In indoor dust, the thermally activated magnetochemical transformation is a combination of the chemical process and transformation of magnetic minerals at high temperature. It affects the change in magnetization of the ferromagnetic phase during the heating as well as the cooling. Before heating, the magnetization (*M* ^BH^) of dust can be schematically described by two ferromagnetic/ferrimagnetic components and the paramagnetic and diamagnetic components

2 $$ {M}^{\mathrm{BH}}={M}_{\mathrm{mag}}+{M}_{\mathrm{Fe}}+{M}_{\mathrm{P}\hbox{-} \mathrm{D}} $$

where *M*_mag_ is the magnetization carried by the magnetite fraction, *M*_Fe_ is the magnetization of iron particles, and *M*_P-D_ is the magnetization of paramagnetic and diamagnetic parts of dust. After heating up to 800 °C and cooling back to room temperature, the magnetization (*M* ^AH^) can be expressed by

3 $$ {M}^{\mathrm{AH}}={M}^{\mathrm{BH}}+\varDelta {M}_{\mathrm{nf}\ \mathrm{m}\mathrm{a}\mathrm{g}}-\varDelta {M}_{\mathrm{Fe}\ \mathrm{oxidation}} $$

where Δ*M*_nf mag_ is the magnetization of the newly formed magnetite by the oxidation of metallic iron and by thermal alteration of non-magnetic minerals and Δ*M*_Fe oxidation_ is the loss of magnetization coming from oxidation of metallic iron to magnetite. It is clear from Eq. (3) that the changes in magnetization depend on the amount of Fe and intensity of the thermally induced chemical processes producing new magnetite.

## Conclusion

The coexistence of magnetite and iron in indoor dust makes its magnetic properties complex. Variable amounts of metallic iron are responsible for the degree of magnetic enhancement expressed by the high value of saturation magnetization and magnetic susceptibility. Moreover, the presence of Fe particles contributing to magnetite influences the shape of the hysteresis loop and its parameters. The heating of indoor dust in air shows several distinct features on the thermomagnetic curves of *M*(*T*) as well as *κ*(*T*), caused by oxidation of metallic iron and neo-formation of magnetite. The presence of metallic Fe appears as a decreasing tail on the heating curve of *κ*(*T*) between 600 and 700 °C and as the Curie temperature of ∼760–765 °C during the samples’ heating up to 800 °C in the magnetization vs. temperature curves. The rapid decrease observed between 600 and 700 °C on the heating curve of *κ*(*T*) is interpreted as the oxidation of certain amounts of iron to magnetite which is effectively visible in the cooling curve coming back from 700 to 580 °C.

The magnetic fraction of the magnetite-Fe mixture was examined to explain how metallic iron affects the distribution of hysteresis ratios on the Day-Dunlop plot. The analysis was conducted based on the changes in the parameters of hysteresis loops measured before and after oxidation of Fe to magnetite. The samples containing a mixture of magnetite and iron locate towards the MD area of the Day-Dunlop plot. The thermal treatment causes the shift of hysteresis ratios from the MD region towards the PSD-SD region. This effect is mostly controlled by the amount of metallic iron and by its oxidation to magnetite during the heating process.

The thermal treatment (the heating to 800 °C and cooling back to room temperature) results in two processes: the oxidation of metallic iron to magnetite and the neo-formation of magnetite as the result of the oxidation of metallic iron and the chemical transformation of non-magnetic minerals (minor process).

Indoor dust has been characterized for environmental magnetism study because of its application as a potential indicator of indoor air pollution. Elemental iron plays an important role in the development of inflammation in humans via oxidative stress, so the presence of metallic Fe in indoor dust can affect human health.
